# Shared Care Uptake for Attention Deficit Hyperactivity Disorder Medication in Hull and East Riding of Yorkshire: An Exploratory Audit

**DOI:** 10.1192/bjo.2026.11575

**Published:** 2026-06-30

**Authors:** Adebusola Adegbola, Karen Thompson, James Donnelly, Stella-Maris Okwukaogu

**Affiliations:** Humber Teaching NHS Foundation Trust, Hull, East Riding Of Yorkshire, United Kingdom

## Abstract

**Aims:**

The National Institute for Health and Care Excellence (NICE) and local prescribing guidance recommend that, after titration and dose stabilisation, prescribing and monitoring of Attention Deficit Hyperactivity Disorder (ADHD) medication should be carried out under shared care arrangements with primary care. In practice, shared care requests are accepted to varying degrees, thereby increasing service burden for secondary care.

We aimed to estimate the current acceptance rate of shared care requests for ADHD medications in Hull and the East Riding, explore factors influencing uptake, and recommend improvements to shared care arrangements with primary care.

**Methods:**

A retrospective exploratory audit was conducted using electronic patient records from the Children and Young People’s Neurodevelopmental Service.

The data capture period was from 01/07/2025 to 01/09/2025. The audit included a consecutive sample of all patients identified.

The patient’s ADHD medication, shared care request, outcome, and reasons for prescription decisions, despite a shared care agreement, were recorded in an Excel spreadsheet. Data were collected retrospectively in December 2025.

**Results:**

Of 114 patients identified, 62 (55%) had shared care requests, 50 (45%) had none, and 2 were not medicated. Of the 62 patients who had shared care requests, 43 (69%) were accepted, 13 (21%) received no response, and 6 (10%) were declined. 4 of the 6 patients whose shared care protocol was declined are from the same General Practitioner (GP) Practice, which cited local prescribing guidelines for declining to prescribe or monitor methylphenidate. There was no clear documentation regarding prescribing decisions despite a shared care agreement in place. Prescribing commonly followed dose changes, formulation changes, or drug holidays. 2 patients were still undergoing titration.

**Conclusion:**

Shared care uptake in primary care could be improved primarily by increasing the rate and timeliness of requests from secondary care, implementing systematic follow-up, and targeting engagement with GP practices that are not responding to or declining shared care. These recommendations, along with routine documentation of shared care status in clinic letters and a clear rationale for prescribing despite a shared care agreement, have been communicated to all prescribers in the service and other relevant stakeholders. A re-audit is planned to assess the impact of the implemented recommendations on improving uptake, facilitating easier access to medication, reducing service burden, and increasing capacity for new patients.

**Acknowledgements**

We thank:

Dr Syed S. M. H. Naqvi, Consultant Child and Adolescent Psychiatrist**,** for supervision and guidance.

Jo Allison, Nurse Prescriber, for data support.

